# Water-Soluble Formulations of Curcumin and Eugenol Produced by Spray Drying

**DOI:** 10.3390/ph18070944

**Published:** 2025-06-23

**Authors:** Iskra Z. Koleva, Katya Kamenova, Petar D. Petrov, Christo T. Tzachev

**Affiliations:** 1 Faculty of Chemistry and Pharmacy, Sofia University “St. Kliment Ohridski”, 1 J. Bourchier Blvd., 1164 Sofia, Bulgaria; ohtct@chem.uni-sofia.bg; 2 Institute of Polymers, Bulgarian Academy of Sciences, “Akad. G. Bonchev” Street, Bl. 103A, 1113 Sofia, Bulgaria; ppetrov@polymer.bas.bg; 3 Centre of Competence “Sustainable Utilization of Bio-Resources and Waste of Medicinal and Aromatic Plants for Innovative Bioactive Products” (CoC BioResources), 1000 Sofia, Bulgaria

**Keywords:** curcumin, eugenol, Soluplus^®^, double-loaded formulations, encapsulation, low-temperature spray drying, green technology

## Abstract

**Background/Objectives**: In this study, we present a green, scalable platform for the production of water-dispersible powders co-encapsulating the lipophilic bioactives curcumin (Cur) and eugenol (Eug) within the amphiphilic polymer Soluplus^®^ (SP) via low-temperature spray drying. **Methods**: The amount of Cur (1%, 5%, and 10%) and Eug (5%, 10%, 15%, and 20%) was varied to achieve single- and double-loaded water-soluble powders with the maximum amount of active substances. The powders containing a higher loading of Cur, 5% and 10% (and Eug), were obtained from water/ethanol mixtures (2:1 and 5:1 *v*/*v* ratio), while the formulation with 1% of Cur was spray-dried by using water as a solvent. **Results**: By leveraging aqueous or aqueous–ethanolic feed systems, we achieved high loading of the bioactive substances—up to 10% Cur and 20% Eug (*w*/*w*)—while minimizing organic solvent use. Myo-inositol was incorporated as a stabilizing excipient to modulate particle morphology, improve powder flowability, and enhance redispersibility. Physicochemical characterization revealed nanoscale micellization (53–127 nm), amorphization of both actives as confirmed by XRD and DSC, and the absence of crystalline residue. Encapsulation efficiencies exceeded 95% for Cur and 93% for Eug. Dissolution tests demonstrated a rapid release from the 5% Cur/5% Eug formulation (>85% in 5 min), while higher-loaded single-formulations showed progressively slower release (up to 45 min). **Conclusions**: This work demonstrates a robust and environmentally responsible encapsulation strategy, suitable for delivering poorly water-soluble phytochemicals with potential applications in oral nutraceuticals and pharmaceutical dosage forms.

## 1. Introduction

Curcumin (Cur) and eugenol (Eug) are natural bioactive substances possessing antioxidant, antifungal, antibacterial, anti-inflammatory, and anticancer properties [1,2,3,4,5,6,7,8,9,10,11,12,13,14,15,16,17,18,19,20]. In particular, Cur is a crystalline substance with a bright orange–yellow color. It is one of the main constituents in the roots of *Curcuma longa* L., known in Asian cuisine as the spice turmeric [21]. Cur finds applications in food colorings and food additives. Eug is a light-yellow oil, which was first isolated in 1826 from cloves. Eug is used in pharmaceuticals, cosmetics, dentistry, the food industry, and agriculture as a pesticide [22,23]. Curcumin and eugenol have demonstrated significant potential for synergistic therapeutic activity. Recent studies suggest that combining curcumin’s anti-inflammatory and antioxidant properties with eugenol’s complementary antimicrobial and analgesic effects could enhance their pharmacological efficacy [24,25]. Co-encapsulation of both substances in a water-soluble formulation may thus provide improved bioavailability and an expanded therapeutic profile compared to their individual applications. However, both compounds are poorly water-soluble, limiting their bioavailability. The solubility of Cur and Eug in water can be increased by embedding them into polymer micelles through hydrophobic interactions [26,27,28]. Soluplus^®^ (SP) is a graft copolymer composed of a hydrophobic part made of polyvinyl caprolactam and polyvinyl acetate and a hydrophilic segment of polyethylene glycol. SP is a copolymer with amphiphilic properties that forms nanosized structures in water. SP-based drug delivery systems, such as micelles, solid dispersions, nanosuspensions, and nanoemulsions, are widely investigated for improving the solubility and bioavailability of various therapeutic agents [29]. Spray drying is considered a green method for preparing powders that have fast solubility [30]. It is a rapid one-step method appropriate for temperature-sensitive compounds with an industrial application in pharmacy, chemistry, and food preparation [31,32,33,34,35,36,37]. Kamal et al. developed a curcumin-loaded powder by spray drying using SP and Neusilin^®^ as solid carriers and ethanol as a solvent [38]. They formulated a self-emulsifying drug delivery system (SEDDS) by dissolving curcumin in sulforaphane, followed by mixing with cremophor EL and acconon CC6 (1:1 (*w*/*w*) mixture). The obtained powders, which were determined as the optimal composition with good flow characteristics, contained 0.9% of Cur based on the mass of the polymer. Other research also reported encapsulation of Cur using spray drying in ternary-composite wall materials like β-cyclodextrin, modified corn starch, maltodextrin, gelatin, pectin, tamarind gum, and inulin [39]; soy protein isolate nanocomplexes [40]; gum arabic [41]; gum arabic, sodium alginate and modified chitosan [42]; and maltodextrin, whey protein isolate and HI-CAP^®^100 (modified starch) [43].

Al-Akayleh et al. also investigated the effect of the solubility and dissolution rate of Cur using SP [44]. They reported a significant improvement in the solubility and in vitro release of curcumin. Besides the spray drying method, Cur-loaded Poloxamer^®^ 188 and SP nanoparticles were obtained by antisolvent crystallization followed by freeze drying [45]. Curcumin-loaded mixed Soluplus/D-α-tocopheryl polyethylene glycol succinate (TPGS 1000) micelles were also reported [46]. Similarly, SP, Syloid^®^ XDP 3150, Syloid^®^ 244, and Poloxamer^®^ 188 in combination with HPMC E5 (binary carrier) dispersions containing curcumin using the solvent evaporation method were prepared [47]. Another study also examined the interaction of SP with curcumin using UV/VIS, fluorescence, and infrared spectroscopies [48].

Using the spray drying method, Eug was encapsulated in various carriers such as maltodextrin and gum arabic, soy lecithin and whey protein, as well as rice bran protein, albumin, and carrageenan [49,50,51]. In a previous study, we obtained Eug-loaded Soluplus^®^ and Lutrol F 127 spray-dried powders starting from a water dispersion [28]. We optimized the flowability of the formulations by the addition of myo-inositol (cyclohexanehexol isomer—a sweetener, hydrophilizing, and powder structure-forming agent) and Aerosil^®^ 200 (amorphous colloidal silica—thermal stabilizer, pore-forming, anticaking, and powder gliding agent). As far as we know, formulations comprising both Cur and Eug are limited. Cur- and Eug-containing mucoadhesive gel for periodontal disease was recently formulated using Poloxamer 407 and Carbopol 934 [52,53].

However, there are currently no data in the literature on the simultaneous encapsulation of curcumin and eugenol in SP. The main objective of our study is to obtain water-soluble spray-dried powders, containing curcumin and eugenol, based on SP as a carrier. Although using water as a solvent is preferable, it was not possible to obtain stable feed solutions for spray-drying that contain more than 1% curcumin. Therefore, a mixture of water and ethanol was used for the high concentrations of the active ingredient(s). Combining water with water-miscible solvents such as ethanol is advantageous for improving the solubility of poorly water-soluble therapeutic agents, as well as for adjusting the final boiling point, thereby enabling spray drying at lower temperatures [54]. We minimized the use of organic solvents, which, on the one hand, guarantees an environmentally friendly approach and, on the other, makes the process more economically viable.

Optimization of the conditions for preparing Cur/Eug-loaded powders was performed by varying the amount of active ingredients, the water/ethanol ratio (2:1 and 5:1), and the concentration of the spray drying feed solutions. The ultimate goal of the series of experiments was to develop powders containing the maximum amount of active ingredients, which are water-soluble and form stable Cur/Eug-loaded colloidal nanoparticles upon dissolution in water. Several studies have reported the impressive benefits of inositol intake, such as alleviating symptoms of polycystic ovary syndrome, lowering serum insulin levels, and improving insulin resistance approximately twice as well as pioglitazone or metformin, which are used as therapies for patients with glucose intolerance [55]. Therefore, as in our previous study [28], we optimized the composition by the addition of myo-inositol to improve the flowability and solubility of the powders.

The powders were characterized using scanning electron microscopy (SEM), Fourier-transform infrared spectroscopy (ATR-FTIR), X-ray diffractometry (XRD), and differential scanning calorimetry (DSC). The zeta potential, particle size, and dispersity index of the colloidal solutions after dissolving the powders in water were determined by dynamic light scattering (DLS). UV–VIS spectroscopy was used to evaluate the amount of encapsulated curcumin and eugenol.

## 2. Results

Spray-dried single- and double-loaded powders containing Cur and Eug were prepared using the amphiphilic block copolymer SP. Optimization of the Cur- and Eug-loaded spray-dried powders was performed by varying the amount of curcumin (1%, 5%, and 10%) and eugenol (5%, 10%, 15%, and 20%) with respect to the mass of SP (4 g), the water/ethanol ratio (2:1, 5:1 and 1:0), and the concentration of the starting solution of SP (6.7 and 3.3% *w*/*v* for Cur-loaded powders, 10 and 6.7% *w*/*v* for Eug-loaded powders, and 3.3% *w*/*v* for Cur/Eug double-loaded powders). The spray-drying process parameters were kept constant for all formulations, including a feed rate of 6 mL/min, a nozzle diameter of 0.7 mm, an atomization gas flow rate of 414 L/h, an aspiration rate of 35 m^3^/h, and an inlet temperature of 80 °C. The detailed composition of the feed dispersions used for spray drying is provided in Table S1 of the Supplementary Materials.

### 2.1. Effect of Solvent on Polymer Aggregation

Preliminary studies of SP aggregation in water, ethanol, and water/ethanol mixture (1 gL^−1^) were conducted by DLS analysis (Supplementary Materials, Figure S1). SP forms polymer micelles in pure water with an average hydrodynamic diameter (D_h_) of about 63 ± 4 nm, while in ethanol, the polymer does not aggregate at the reported concentration [56]. Dissolving SP in a mixed solvent led to the formation of particles of different sizes, depending on the composition of the water/ethanol mixture. At a higher water content (5:1 *v*/*v* ratio), the particles have a D_h_ of 140 nm, and with the increase in the portion of ethanol (2:1 *v*/*v* ratio), relatively large particles/agglomerates with a D_h_ of about 1000 nm were observed.

### 2.2. Preparation and Optimization of Curcumin-Loaded Nanoparticles and Spray-Dried Powders

Firstly, we investigated the incorporation of Cur into SP micelles obtained in aqueous solution and water/ethanol mixture. For comparison, a sample in ethanol solution (only for the high concentration of curcumin, 10%) was also prepared. Thus, formulations (powders) containing 1% Cur (1%C-S), 5% Cur (5%C-S), and 10% Cur (10%C-S) with respect to the mass of SP were prepared by adding Cur to the micellar solutions and subsequent spray-drying. The concentration of the initial polymer solution was 6.7 and 3.3% *w*/*v*. Next, the obtained powders were dissolved in pure water (1 gL^−1^), and the samples were characterized by dynamic and electrophoretic light scattering. The parameters such as the yield, hydrodynamic diameter, particle size distribution, and zeta potential of the Cur-loaded micelles are given in Table 1.

**Figure 1:**
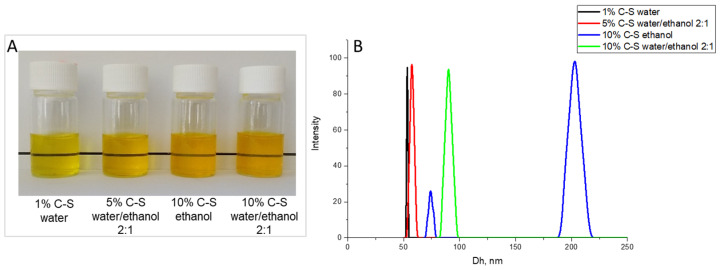
Photographs (**A**) and hydrodynamic diameter (**B**) of aqueous solutions of Cur-loaded SP nanoparticles containing 1% Cur (1%C-S), 5% Cur (5%C-S), and 10% Cur (10%C-S), prepared using water for 1%C-S, a water/ethanol mixture at a 2:1 ratio for 5%C-S and 10%C-S, and ethanol for 10%C-S, with the starting solution containing 6.7% *w*/*v* SP.

The yield of the powders loaded with 1% curcumin, 1%C-S, was approximately 30%. After dissolution in water, nanoparticles with a D_h_ of 53 nm and a negative zeta potential of −3.8 mV were found. The corresponding parameters of the unloaded SP powder are 54 nm and −3.0 mV (Supplementary Materials, Figure S2). The spray-dried formulations with 5% curcumin, prepared from a starting aqueous solution, had a yield of about 22%, and after dissolution in water, a curcumin precipitate formed. This result clearly shows that under these conditions, the poorly water-soluble Cur was not completely loaded into the nanocarriers, resulting in precipitate with low water solubility. Adding a small amount of ethanol to the starting solution (2:1 water/ethanol) resulted in stable colloidal particles after dissolution in water, whose average hydrodynamic diameter was 57 nm (Figure 1), but the resulting powder had a low yield of 24.7%.

The powder obtained from an unstable starting aqueous solution of 10% curcumin is in low yield and forms a precipitate after addition to an aqueous medium. The yield of the powders obtained from a pure ethanol solution significantly increases to 52.5%, but when dissolved in water, the particles have a bimodal distribution, with a D_h_ of 74 nm and 204 nm, respectively. The formulations obtained from a water/ethanol mixture showed a monomodal distribution of the particles after dissolution of the powders with an average hydrodynamic diameter of 90 nm.

**Figure 2:**
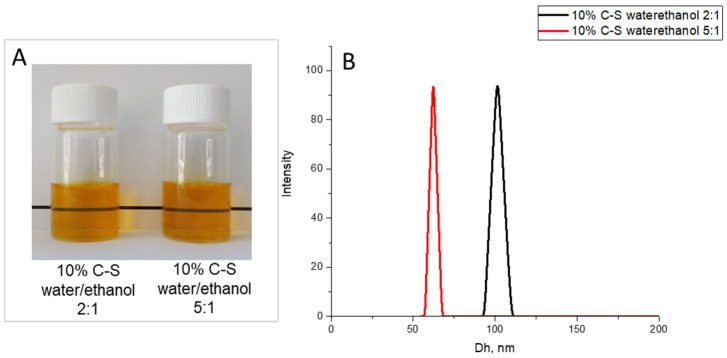
Photographs (**A**) and Hydrodynamic diameter (**B**) of aqueous solution of Cur-loaded SP nanoparticles, containing 10% Cur (10%C-S) obtained at various water/ethanol ratio (2:1 and 5:1) of the starting solution at 3.3% *w*/*v* of SP.

The concentration of the starting polymer solution is one of the parameters that influence the properties of the final product [54,57]. The effect of the concentration of feed solution was evaluated at two different concentrations: 6.7 and 3.3% *w*/*v* (Table 1). All powders obtained from the water/ethanol mixture (2:1 and 5:1) formed stable colloidal dispersions. The particle size at 2:1 water/ethanol ratios was approximately 100 nm, while for the powders obtained at 5:1 water/ethanol ratios, the average hydrodynamic diameter of the colloidal particles decreased to 62 nm (Figure 2). It is important to note that decreasing the SP concentration led to an increase in the yield to above 50%. Powders prepared at the same water/ethanol ratio (2:1) but at different Cur concentrations showed a significant difference in the yield of the final product. The yield of spray-dried formulations at 3.3% *w*/*v* was twice as high as compared with the more concentrated solution (6.7% *w*/*v*), while the difference in nanoparticle size was insignificant.

### 2.3. Preparation and Optimization of Eugenol-Loaded Nanoparticles and Spray-Dried Powders

In our previous study, spray-dried eugenol carriers were developed using the polymers Soluplus^®^ and Lutrol F 127, with feed solutions prepared in water at polymer concentration 10% *w*/*v* [28]. The incorporation of eugenol (5%, 10%, 15% of the total mass of the polymers) at different ratios of polymers to active substance was investigated. In the present study, we prepared powders containing 20% Eugenol with respect to the mass of SP. For comparison with the previously obtained formulations [28] and some of the curcumin-containing powders, the solubilization of 20% eugenol was investigated by varying the SP concentration of the starting solution (10 and 6.7% *w*/*v*) by preparing the dispersions in water and a 2:1 water/ethanol mixture. The obtained spray-dried powders were dissolved in water, and the colloidal solutions (1 mg/mL) were characterized by DLS measurements. The results are presented in Table 2.

**Figure 3:**
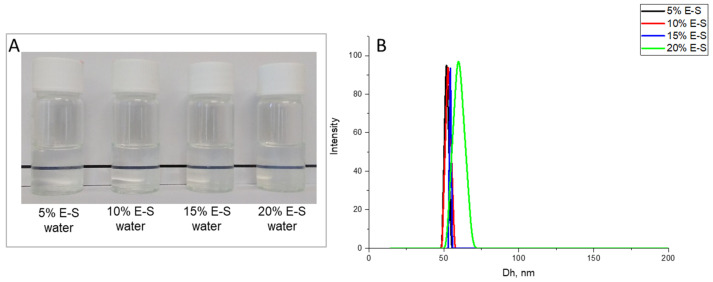
Photographs (**A**) and hydrodynamic diameter (**B**) of aqueous solution of Eug-loaded SP nanoparticles, containing 5% Eug (5%E-S), 15% Eug (15%E-S), and 20% Eug (20%E-S) obtained at a starting aqueous solution at a 10 mass % of SP.

The hydrodynamic particle diameters of spray-dried samples with different percentages of eugenol (5%, 10%, 15%, and 20% in aqueous solution at 10% *w*/*v* SP) were approximately 51 nm, 52 nm, 54 nm, and 59 nm, respectively (Figure 3). No significant difference in particle size was observed with increasing the amount of eugenol in the formulations. The yield of the powders of the different formulations varied from 30 to 45%.

**Figure 4:**
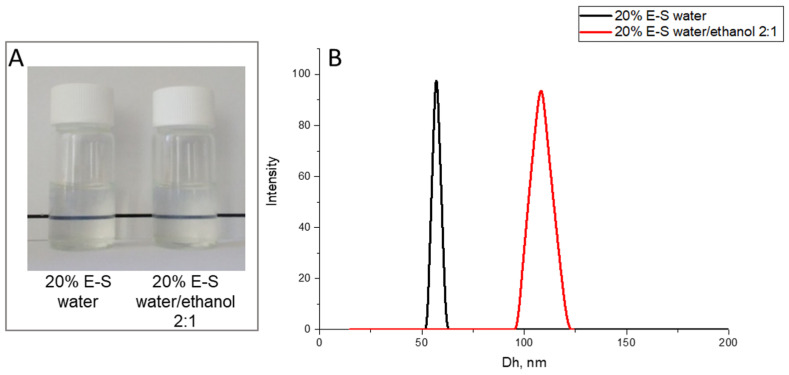
Photographs (**A**) and hydrodynamic diameter (**B**) of aqueous solution of Eug-loaded SP nanoparticles, containing 20% Eug (20%E-S), obtained at a starting solution of water and 2:1 water/ethanol mixture at a 6.7% *w*/*v* of SP.

The preparation of Eug-loaded particles by spray drying was investigated at a lower concentration of SP (6.7% *w*/*v*), using water and a 2:1 water/ethanol mixture as solvent (60 mL). At a decreased concentration of the starting aqueous solution, the hydrodynamic diameter of the particles was almost identical to the particle size obtained from a more concentrated SP solution. The addition of a small amount of ethanol at the same initial volume of the solution led to stable colloidal particles after dissolution of the powder, the size of which increased from 56 nm to 110 nm (Figure 4).

### 2.4. Preparation and Optimization of Curcumin/Eugenol Double-Loaded Nanoparticles and Spray-Dried Powders

Curcumin and eugenol double-loaded powders were prepared by the spray-drying of samples at a curcumin concentration of 5% and 10% and eugenol concentration of 5%, 10%, 15%, and 20%, with respect to the polymer mass. The initial polymer concentration in the feed solutions for spray drying of all studied formulations was 3.3% *w/v*, with a total solvent volume of 120 mL at a water/ethanol ratio of 2:1. The resulting powders were dissolved in deionized water, followed by DLS measurements to determine the particle size and zeta potential. The results are shown in Table 3.

**Figure 5:**
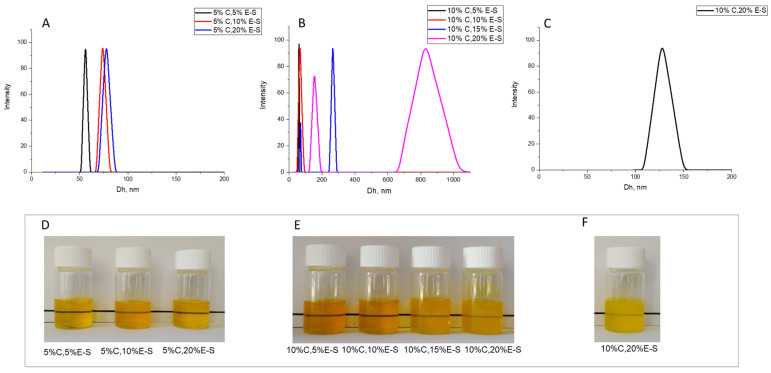
Hydrodynamic diameter and photographs of aqueous solutions of double-loaded curcumin/eugenol SP nanoparticles containing (**A**,**D**) 5% Cur and different amounts of Eug (5%, 10%, and 20%), (**B**,**E**) 10% Cur and different amounts of Eug (5%, 10%, 15%, and 20%), prepared at a water/ethanol ratio of 2:1 (3.3% *w*/*v* SP), and (**C**,**F**) 10% Cur and 20% Eug (10%C,20%E-S), prepared at a water/ethanol ratio of 5:1 (3.3% *w*/*v* SP).

For nanoparticles containing 5% curcumin, an increase in particle size was observed with increasing Eug content. The average hydrodynamic diameter of the particles loaded with 5% Cur and 5% Eug was 56 nm, and at 5%C-S, 10%E-S and 5%C,20%E-S, the size increased to 76 nm and 78 nm, respectively (Figure 5A). The yield of powders decreased with increasing eugenol content in the starting solution.

The formulations containing 10% curcumin (Figure 5B) showed a negligible effect on particle size at 5% and 10% eugenol. The sizes were similar to the colloids loaded with 5%C,5%E-S and 5%C,10%E-S. Increasing the amount of eugenol to 15% and 20% led to an increase in particle size and the appearance of a second peak at 260 nm and 800 nm, indicating the presence of larger particles/aggregates. It is likely that under these conditions (water/ethanol ratio), a larger amount of the active substances remained out of the SP matrix during spray drying, which, after dissolution of the powders, led to an unstable colloidal solution.

This result necessitated a change in the ratio of the two solvents. 10%C,20%E-S powders in a 5:1 water/ethanol mixture were prepared to investigate the possibility of loading the two active substances into an SP carrier and obtaining stable colloidal dispersions after dissolution in water. The results show that changing the solvent ratio leads to the formation of stable colloidal solutions without any precipitation after dissolution of the powder in an aqueous medium. The particles have a monomodal size distribution and an average hydrodynamic diameter of 127 nm (Figure 5C). Photographs of all micellar solutions are presented in Figure 5D–F.

**Figure 6:**
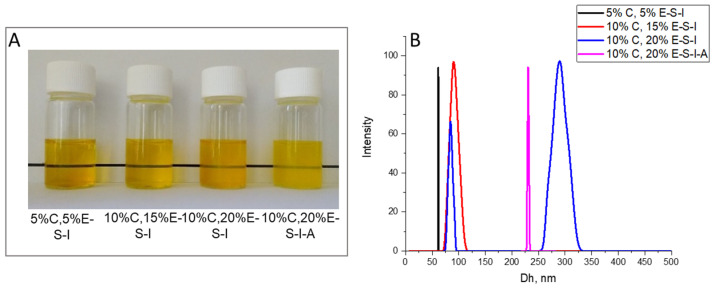
Photographs (**A**) and hydrodynamic diameter (**B**) of aqueous solution of double-loaded curcumin/eugenol SP nanoparticles in the presence of myo-inositol and/or Aerosil^®^ 200.

To improve the characteristics of the powders, myo-inositol and/or Aerosil^®^ 200 were added to the feed solutions of double-loaded SP nanoparticles. This resulted in a larger average hydrodynamic diameter of 61–230 nm. Sample 5%C,5%E-S-I D_h_ slightly increased by 5 nm compared to 5%C,5%E-S and the size distribution remained narrow. The formulations with a higher amount of curcumin and eugenol exhibited a more significant increase in D_h_ (up to 103 nm) and in some cases, the distribution became bi- or multimodal (10%C,20%E-S-I and 10%C,20%E-S-I-A) (Figure 6).

### 2.5. Appearance and Morphology of the Spray-Dried Powders

**Figure 7:**
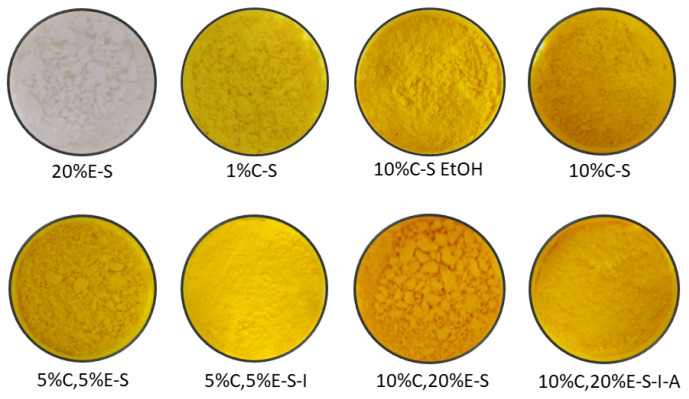
Selected spray-dried powders.

The spray-dried powders are yellow colored in the case of the curcumin-containing formulations (Figure 7). The increase in the eugenol content leads to aggregation of the particles (compare 5%C,5%E-S and 10%C,20%E-S powders). The 20%E-S formulation is white in color, prone to the same adhesion of the particles as the double-loaded compositions with eugenol. The powder containing 1% curcumin was obtained only from a water solution, and some aggregation of the particles is also observed. Myo-inositol reduces the aggregation process, and the powders are finer with improved flowability.

**Figure 8:**
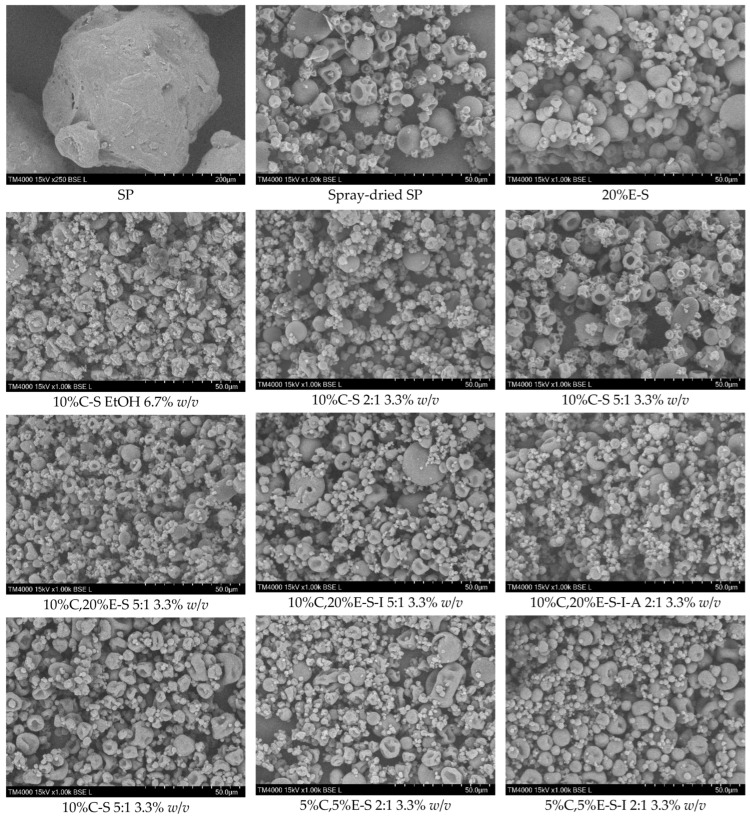
SEM micrographs of selected powders.

The powder’s morphology was studied via scanning electron microscopy (SEM). After spray drying, the solid particle size of SP was significantly reduced from around 300 μm to 10 μm or even less (Figure 8). The SEM micrograph of the unloaded spray-dried SP shows differences in the size of microparticles, which are characterized by a spherical shape and a pronounced concavity. This morphology indicates that the particles are rather hollow and collapse upon solvent removal due to deformation forces. Such structure is visible also in the single-loaded and double-loaded formulations. In the case of the powder containing only eugenol, 20%E-S, less deformed spherical particles are observed. The addition of myo-inositol also affects the deformation of the particles and minimizes their collapse, while the presence of Aerosil^®^ 200 does not seem to change the morphology of the particles further. When ethanol is used as a solvent, the shape of spray-dried solid particles was more flattened, compared to the particles obtained from water or a water/ethanol mixture (Figure 8, 10%C-S EtOH 60 mL). For comparison, in the Supplementary Materials Figure S3, SEM micrographs of the pure curcumin and other selected formulations are shown, including the 20%E-S sample obtained using the 2:1 water/ethanol ratio of the solvents. Interestingly, in this case, the morphology is drastically different as no individual particles are observed.

### 2.6. Encapsulation Efficiency and Loading Capacity

**Figure 9:**
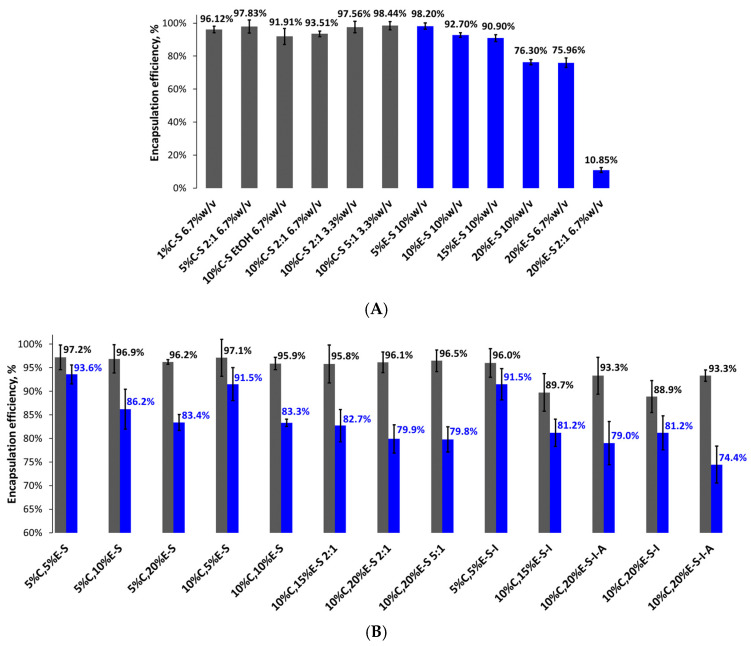
Encapsulation efficiency of single-loaded curcumin (1%C-S, 5%C-S and 10%C-S) and eugenol (20%E-S) powders (**A**). For comparison, the results from our previous study of the 5%, 10%, and 15%-S formulations are also shown [28]; double-loaded curcumin and eugenol formulations (**B**). Results for eugenol are shown in blue and for curcumin in dark gray.

The encapsulation efficiency (EE) in the case of the 20% eugenol-loaded powders shows that around 76% of the eugenol is successfully entrapped in the polymer (Figure 9). However, this result is superior concerning the EE of 15%E-S powder since the total mass of the entrapped eugenol is higher by 61 mg and actually corresponds to 15% eugenol mass to the mass of the polymer. The encapsulation efficiency of the powder obtained from the dispersion containing 2:1 water/ethanol ratio is only 11%. It should be noted that although in the feed dispersion for spray-drying no precipitation was observed, during the spray drying process, the cyclone separator was covered by a significant amount of the polymer.

The encapsulation efficiency of the curcumin-loaded formulations varies from 91.9% to 98.4% for 10%C-S (obtained from ethanol solution) and 10%C-S (feed dispersion water/ethanol 5:1), respectively (Figure 9). The EE in the case of 1%C-S powder obtained from a water solution is 96.1%. The EE in the case of the 10%C-S powder obtained from twice as concentrated feed dispersion (6.7% *w*/*v* water/ethanol 2:1) is lower by 4.1% compared to that obtained from 3.3% *w*/*v* water/ethanol 2:1 dispersion. 

For most of the double-loaded powders, the EE of curcumin is roughly around 95% (see Figure 9) and that of eugenol is above 80%. The highest EE for curcumin and eugenol was found in the case of 5%C,5E-S formulation, 97.2% and 93.6%, respectively. In general, as could be expected, the eugenol encapsulation is higher (~90%) in the powders obtained from dispersions containing 5% eugenol with respect to the SP mass, compared to those prepared from the dispersions with a higher eugenol concentration of 20% (EE ~80%).

**Figure 10:**
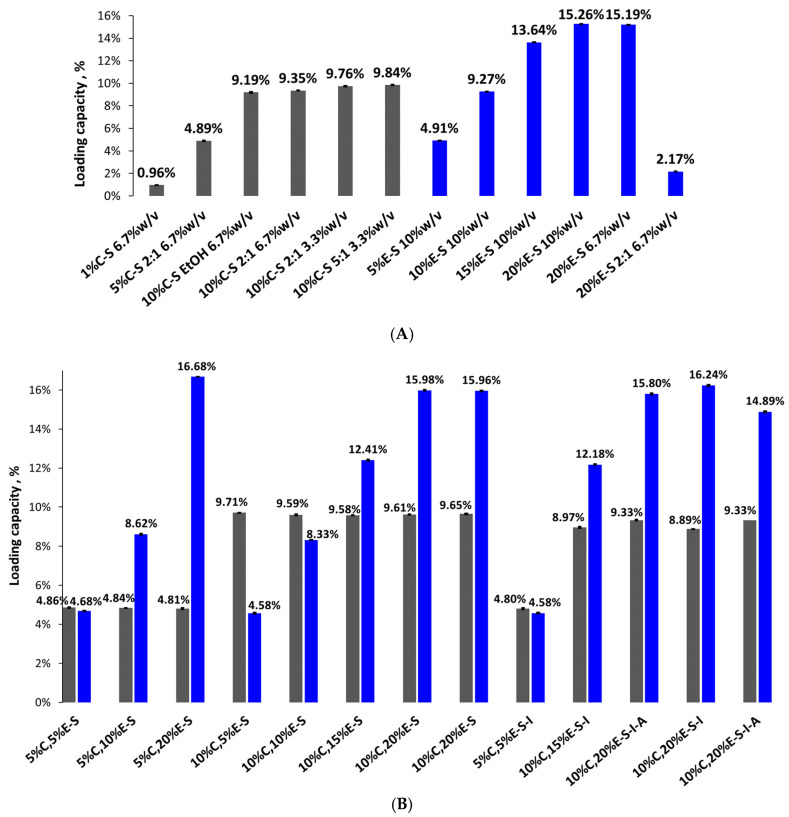
Loading capacity of single-loaded curcumin (1%C-S, 5%C-S, and 10%C-S) and eugenol (20%E-S) powders (**A**). For comparison, the results from our previous study of the 5%-S, 10%-S, and 15%-S formulations are also shown [28]; double-loaded curcumin and eugenol formulations (**B**). Results for eugenol are shown in blue and for curcumin in dark gray.

The loading capacity (LC) of eugenol in most cases is higher compared to that of curcumin, due to its higher concentration in the feed dispersions of spray drying (Figure 10). For single-loaded curcumin powders it is in the range of 0.96–9.84% and is highest in the case of 10%C-S formulation obtained from feed dispersion water/ethanol 5:1. Single-loaded eugenol powders show LC from 2.17% to 15.26% as the latter is in the case of 20%E-S formulation (feed dispersion 10% *w*/*v*).

For the double-loaded powders, the LC of curcumin varies from 4.80% to 9.71% for 5%C,5%E-S-I and 10%C,5%E-S, respectively, and that of eugenol is in the range 4.58–16.68% for 10%C,5%E-S/5%C,5%E-S-I and 5%C,20%E-S, respectively.

### 2.7. FTIR Analysis

**Figure 11:**
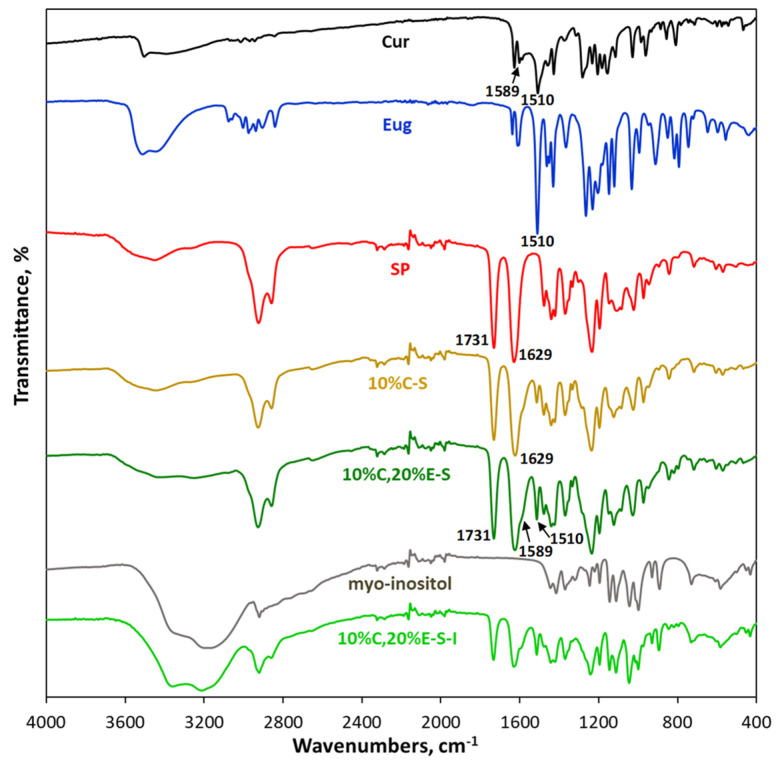
ATR-FTIR spectra of selected powders and pure compounds.

In the FTIR spectra of both curcumin and eugenol, a band at around 1510 cm^−1^ is observed, assigned to the ring stretching vibrations, ν(C = C). Such a band is absent in the spectrum of the pure SP and myo-inositol but is visible in the spectrum of the spray-dried samples, 10%C-S, 10%C,20%E-S and 10%C,20%E-S-I, confirming the presence of the loaded substances (Figure 11). Since all of the studied compounds have OH groups forming hydrogen bonds, the typical broad band of O-H stretching vibrations is visible in the 3600–3200 cm^−1^ region of the spectra. In the spectrum of curcumin, a sharp band at 3508 cm^−1^ is observed due to the presence of free OH groups, while the wide band in the region 3500–3130 cm^−1^ is attributed to OH groups forming hydrogen bonds. The stretching vibrations at 1628 cm^−1^ are assigned to the curcumin carbonyl groups. The band is significantly shifted to lower wavenumbers not only because the carbonyl groups are conjugated with the neighboring C = C bonds but also most likely because of the enol form in which an intramolecular hydrogen bond is formed [45]. The band at 1259 cm^−1^ could be attributed to the enol C-O stretching vibration [45]. In the spectrum of eugenol, absorption bands at 3600–3200 cm^−1^ of the hydrogen-bonded OH groups and 1611 cm^−1^ and 1510 cm^−1^ stretching vibrations of the aromatic ring and v(C-O) at 1265 cm^−1^ of the phenol group are observed, consistent with previous studies [28,51].

The ester and the amide carbonyl groups of the unloaded SP absorb at 1731 cm^−1^ and 1629 cm^−1^, respectively, also in line with previous research [28,44,45,47]. There is no significant shift of these bands in the spectra of the single- and double-loaded formulations, implying that the carbonyl groups do not participate in hydrogen bonds with the loaded substances. The shoulder at 1589 cm^−1^, which partially overlaps with the amide carbonyl band of SP, observed in the spectra of the single- and double-loaded powders, is assigned to the C = C stretching vibrations of the aromatic rings of curcumin.

Since there is no notable difference between the spectra of the spray-dried samples and pure compounds, it can be concluded that no strong interactions or covalent bonds occurred between the polymer matrix and the loaded substances. Ishtiaq et al. also concluded, based on the FTIR analysis, that there is no chemical bonding between Cur and SP [47].

### 2.8. Differential Scanning Calorimetry Analysis

**Figure 12:**
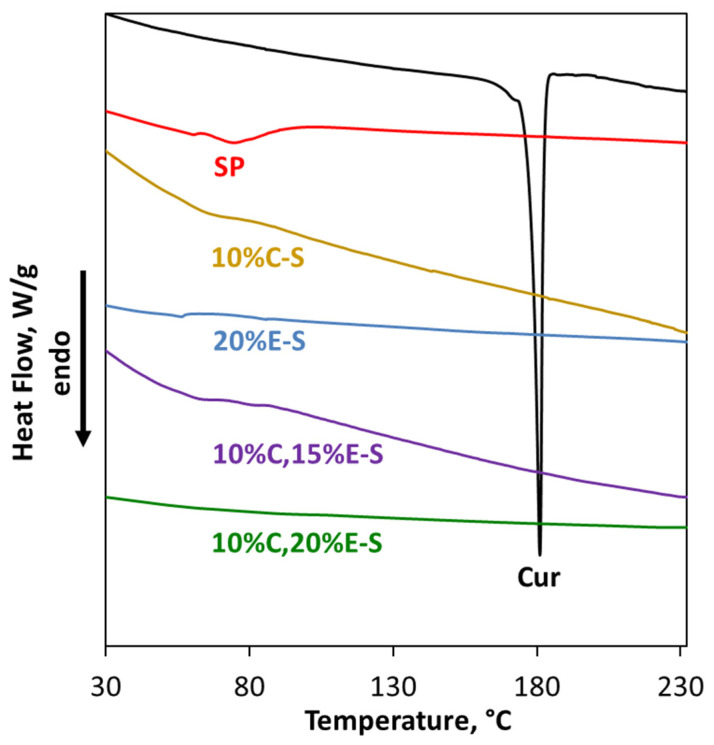
DSC thermograms of Cur, SP, and selected powders. The 10%C-S and 10%C,20%E-S are obtained from the 5:1 water/ethanol ratio dispersion.

Differential scanning calorimetry (DSC) analysis was employed to evaluate the thermal behavior of pure Cur, SP, and their binary and ternary formulations (Figure 12). The DSC thermogram of Cur exhibits a sharp endothermic peak at 181 °C, corresponding to its melting temperature. The peak is absent in the thermograms of the spray-dried single- (1%C-S and 10%C-S) and double-loaded (10%C,20%E-S) formulations containing Cur, implying an amorphous state of the loaded Cur. The absence of the characteristic peaks for the phase transition of curcumin may also suggest that they are embedded within the polymer matrix.

The DSC analysis of as-supplied SP revealed a glass transition temperature (Tg) of 66 °C. This result is consistent with the value stated by BASF-Tg~70 °C [56]. The absence of any melting peak confirms the amorphous state of SP.

### 2.9. X-Ray Analysis

**Figure 13:**
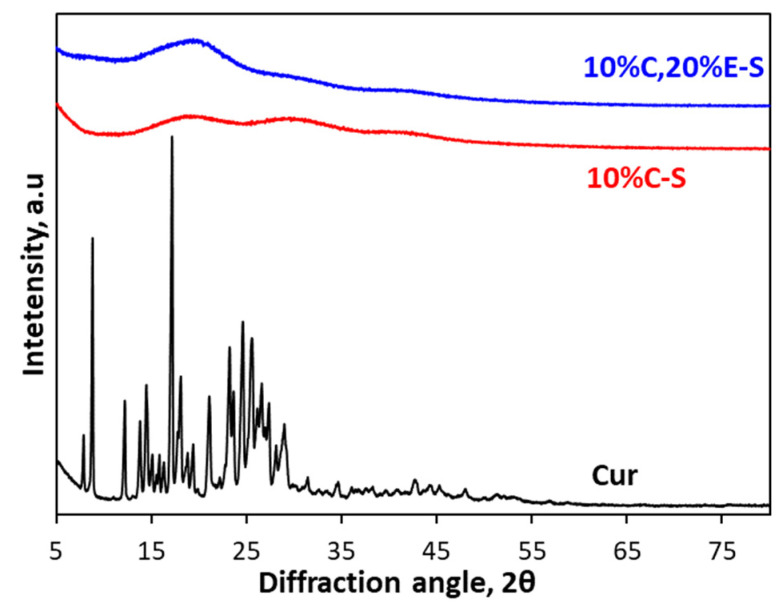
X-ray diffractograms of the pure curcumin (black) and Cur-loaded powder (10%C-S) (red) and Cur and Eug double-loaded powder (10%C,20%E-S) (blue).

XRD analysis was used to investigate the crystal transformation of curcumin before and after encapsulation into spray-dried powders. XRD patterns of pure curcumin, Cur-loaded (10%C-S), and Cur and Eug double-loaded (10%C,20%E-S) powders are shown in Figure 13. The analysis of pure Cur showed many well-defined, sharp characteristic diffraction peaks between 10° and 30°, indicating the crystal structure of the sample. The absence of such well-defined peaks in the diffractograms of the single-(10%C-S) and double-loaded (10%C,20%E-S) powders indicates the successful amorphization of the loaded curcumin upon spray-drying. The amorphous state is expected to increase the water solubility and bioavailability of Cur. These results are in line with the DSC analysis [58,59,60].

### 2.10. Dissolving Properties of the Spray-Dried Powders

**Figure 14:**
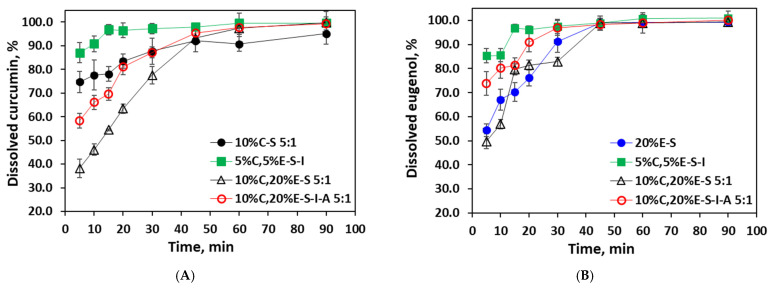
Dissolution profiles of curcumin (**A**) and eugenol (**B**) from selected powders in water at 25 °C and 100 rpm. The data are presented as the mean ± standard deviation, with a sample size of *n* = 3.

The dissolution rate in water of selected powders, 20%E-S (obtained from water dispersion), 10%C-S, 10%C,20%E-S, 10%C,20%E-S-I-A (obtained from water/ethanol 5:1), and 5%C,5%E-S-I (water/ethanol 2:1), was evaluated via the dissolution test (Figure 14). Since the potential application of the powders could be as instant drinks, i.e., before intake to dissolve in a glass of water, the temperature was set to 25 °C. The dissolution speed of the powders is determined by the dissolution rate of the SP microparticles because, in water, eugenol and Cur remain inside the polymer nanoparticles. The highest dissolution rate is observed in the case of the 5%C,5%E-S-I powder, as at the 5th minute, above 85% of Cur is dissolved. For the other studied formulations, 10%C-S, 10%C,20%E-S-I-A, and 10%C,20%E-S, at the same minute, curcumin at 74.3%, 58.3%, and 38.2% was dissolved, respectively. The results for the eugenol are similar to at the 5th minute, where 85.3%, 73.8%, 54.4%, and 49.7% of 5%C,5%E-S-I, 10%C,20%E-S-I-A, 20%E-S, and 10%C,20%E-S are dissolved, respectively.

## 3. Discussion

### 3.1. Experimental Results

Water-soluble SP-based powders with a relatively high content of Cur and Eug were successfully produced by the spray-drying method. Although water was preferred as a solvent, we also used the water/ethanol mixture to obtain stable feed solutions for spray-drying of the formulation containing more than 1% curcumin. To optimize the conditions for obtaining the powders, the potential of SP micelles to separately load the two active substances, Cur and Eug, was initially investigated.

**Figure 15:**
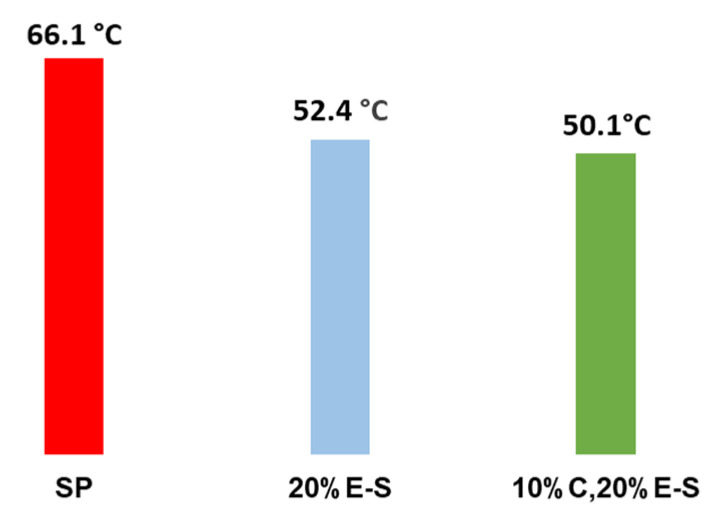
Comparison between the glass transition temperature (Tg) of the pure SP and selected powders containing eugenol (20%E-S and 10%C,20%E-S).

The yield for the single Eug-loaded formulations varies from 37.0 to 47.1%, when only water was used as a solvent for the spray drying dispersions. In the case of water/ethanol mixtures, unexpectedly, the yield was drastically reduced to 12.2% (EE = 11%), due to the fact that a huge amount of the particles were stuck on the cyclone separator of the spray drying apparatus. It was found that the Tgs for the Eug-containing spray dried powder (20%E-S and 10%C,20%E-S) are notably shifted to lower values compared to the neat SP (Figure 15 and Figure S4). This shift could be a sign of the pronounced interaction between the Eug and polymer macromolecules, leading to a plasticizing effect. Such plasticization is consistent with established polymer science principles, whereby the incorporation of small, non-volatile molecules typically enhances polymer chain mobility, resulting in a decreased Tg and increased amorphous flexibility. This effect likely contributes to improved dissolution profiles and enhanced stability of the encapsulated bioactives in aqueous solutions.

On the other hand, different values of the outlet temperature were measured when water, water/ethanol, and ethanol were used as solvents. The increase in ethanol fraction in the feed solution resulted in a rise of the outlet temperature from 41 ± 2 to 48 ± 2 °C. Since the powders containing Eug possess Tg close to the outlet temperature, this makes them more prone to sticking, including on the walls of the cyclone separator. However, it should be noted that these are laboratory experiments, and in industrial conditions, the yield could be increased significantly.

The average hydrodynamic diameter of the Eug-loaded powders (5%, 10%, 15%, and 20%-S powders, Table 2) is around 54 nm, which is practically the same as the average Dh of the 1% curcumin-containing powder of 53 nm. It is very similar to the corresponding diameter of the unloaded spray-dried SP, which is also 54 nm, and to the diameter obtained from Kamal and co-authors for the curcumin-SEDDSs SP—54.33 ± 0.85 nm [38]. The size of the single-Cur-loaded increases with the increase in the curcumin content, which is most likely due to the larger amount of curcumin in the SP micelle cores leading to their expansion (Table 1). However, D_h_ in water solution of the 10%C-S powders obtained from dispersions containing different water/ethanol ratios varies significantly, implying a different number of curcumin molecules in the individual/single micelles—100 nm versus 62 nm, respectively (Table 1). The observed increase in particle size upon addition of ethanol to aqueous SP dispersions can be attributed to reduced solvation of the hydrophilic polyethylene glycol segments of SP. The diminished polarity of the solvent mixture likely lowers the critical micelle concentration (CMC) of SP, facilitating enhanced micelle aggregation and the formation of larger colloidal assemblies. This interpretation aligns with previous studies reporting solvent-dependent aggregation behavior in amphiphilic polymer systems [61,62].

Also, the yield increases with the decrease in the polymer concentration from 26.4% to 56.6%. Usually, a higher solid concentration of the spray drying feed solutions results in an increase in the yield, but in the case of the single-curcumin containing powders, it is exactly the opposite, and the dilution of the dispersion to 3.3% *w*/*v* improves the yield. It was observed that the more concentrated feed dispersions result in more losses in the apparatus, including the feed tube.

**Figure 16:**
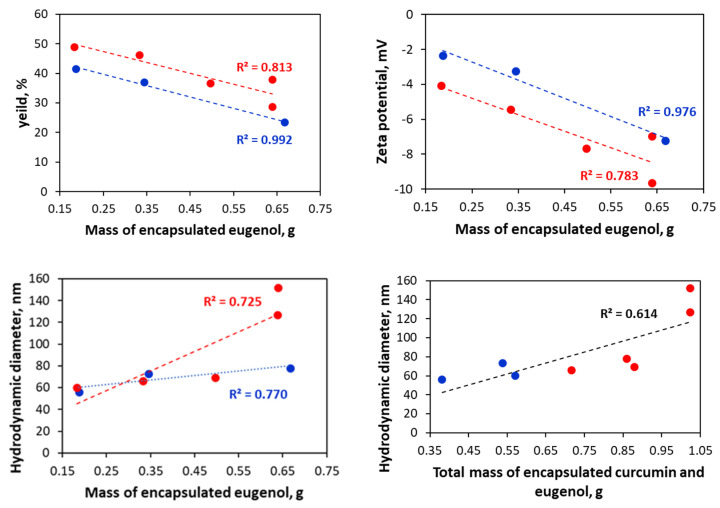
Correlation between yield, hydrodynamic diameter, zeta potential, and eugenol mass and total mass of the encapsulated curcumin and eugenol in the double-loaded formulations. The myo-inositol and Aerosil^®^ 200 containing powders are excluded. The series with formulations named 5%C are shown with blue and 10%C with red.

In the case of the double-loaded formulations, the yield and zeta potential decrease with increasing eugenol content (Figure 16, Table 3). The lowered yield is because, during the spray-drying process, it was observed that some amount of the particles stuck on the cyclone separator of the apparatus, but not as much as in the case of the single-loaded eugenol formulation obtained from the 2:1 water/ethanol ratio. Since the active substances are embedded in the SP micelles, the size of the particles in a water solution increases with the total mass of the curcumin and eugenol in the formulations (Figure 16). Myo-inositol and Aerosil^®^ 200 increase the size of the particles in water solution, as in the case of Aerosil^®^ 200, significantly larger aggregates are observed. This might be due to the formation of hydrogen bonds between the micelles and secondary aggregates.

Myo-inositol also slightly increases the yield from the range 23.4% (5%C,20%E-S)—49% (10%C,5%E-S) to almost 60% since it stabilizes the formation of the powder particles. Although even in the cases of myo-inositol-containing compositions the yield does not exceed 60%, which might not seem a satisfactory yield, one should note that these results are obtained in laboratory conditions and by using a small amount of the substances. Even though in most cases no significant sticking of the polymer on the drying chamber and the cyclone separator of the spray drying apparatus was observed, there are always some losses.

Comparing the SEM micrographs of the powders and the DLS measurements, one may notice that the particle size in the solid state (μm) dramatically differs from the particle size in a water solution (nm), since micelles are formed in water, while the powder particles are formed by the evaporation of the solvent(s), resulting in larger aggregates in which curcumin and eugenol are surrounded by the polymer.

The considerable difference in the behavior of the single-loaded curcumin powders compared to those double-loaded containing a high amount of Eug is due to the aggregation of the particles (see Figure 7, the appearance of the powders), resulting in slower dissolution. Myo-inositol suppresses the aggregation of the particles and is very soluble in water, thus improving the rate of dissolution of the powders.

According to the loading capacity study, with respect to the overall content of eugenol, the double-loaded powders preferably contain a higher concentration of eugenol in the feed dispersion for spray-drying, as 10%C,20%E-S-I could be selected as optimal. However, the LC of the curcumin in this case is not the highest, 8.89%, but this result is satisfying.

Finally, since the obtained powders could find application as food supplements, one should be confident of their safety. Usually, during spray drying, due to the mild conditions (rapid speed of the process and low inlet temperature of 80 °C), no degradation products are expected to occur. Indeed, HPLC studies confirmed the absence of any by-products resulting from the decomposition of the active substances in the powders produced (Supplementary Materials, Figure S5).

### 3.2. Green Technology and Process Optimization

Compared to alternative encapsulation approaches, such as antisolvent crystallization, lyophilization, or encapsulation in protein-based or carbohydrate matrices (e.g., cyclodextrins, maltodextrin, gum arabic), our spray-drying methodology using SP offers significant advantages, including higher loading capacities (up to 10% curcumin and 20% eugenol) and rapid dissolution rates. While lyophilization methods provide a similarly high encapsulation efficiency, spray drying is more advantageous due to its rapid processing time, scalability for industrial application, and minimal use of organic solvents, aligning well with green technology principles. While ethanol was utilized in selected formulations to enhance the solubility of curcumin at higher loadings, formulations such as 1%C-S and 20%E-S were successfully prepared using water as the sole solvent. These water-only systems exemplify a solvent-free approach, thereby eliminating the need for organic solvent recovery, reducing process-related emissions, and lowering production costs—key advantages from both regulatory and environmental perspectives [63,64].

Several challenges intrinsic to aqueous spray drying of hydrophobic compounds were identified and addressed. First, SP exhibits thermoplastic behavior, softening and becoming tacky at elevated inlet temperatures (above 100 °C), which can lead to particle coalescence and wall deposition. This issue was mitigated by employing a relatively low inlet temperature of 80 °C and maintaining low outlet temperatures (41–48 °C), which are compatible with temperature-sensitive actives [65,66]. Second, eugenol’s high volatility posed a risk of loss during drying; hence, reduced processing temperatures and short residence times were critical to retaining the compound within the polymer matrix. Third, the control of residual moisture was essential to achieve optimal flow properties. Excessive moisture can result in plasticized, cohesive powders with poor handling properties, while over-drying may induce electrostatic interactions and particle agglomeration [67,68,69].

To overcome these opposing factors, the polymer concentration, solvent ratio, and drying conditions were carefully tuned. The resulting powders demonstrated moisture levels low enough to maintain powder stability and favorable rheological behavior, while avoiding excessive brittleness or hygroscopicity. Furthermore, the addition of myo-inositol contributed not only to powder structuring and hydrophilization but also improved morphology and dispersibility by reducing particle aggregation and acting as a crystallization inhibitor [70,71].

### 3.3. Implications for Sustainable Manufacturing

These results are consistent with the principles of green formulation science, where aqueous-based spray drying has gained recognition as an environmentally sustainable alternative to solvent-intensive encapsulation techniques [63,64]. SP, although widely used in solubility enhancement, presents formulation challenges due to its thermoplastic nature. As reported by Homayouni et al. [65], spray drying SP above its glass transition (~70 °C) can result in adhesion to drying chamber surfaces and reduced product yield. The use of low inlet temperatures in our study is in line with previous recommendations for temperature-sensitive polymers and bioactives [66].

Eugenol, being a volatile essential oil with a boiling point around 254 °C, is prone to partial evaporation during drying processes. We observed that reducing the drying temperature and minimizing residence time effectively preserved the eugenol content, as reflected in the high encapsulation efficiencies (>90%) in selected formulations.

Moisture control has also been reported as a critical factor in the performance of spray-dried phytochemical powders. As discussed by Shishir et al. [69], residual water levels below 5% are generally required to ensure powder stability and avoid stickiness or caking. The addition of low-molecular-weight excipients such as inositol has been previously shown to improve powder dispersibility and inhibit recrystallization, particularly in sugar-free or protein-free matrices [70,71].

Thus, the formulation strategy adopted here not only achieves high-performance encapsulation but does so using a minimal-solvent, low-energy, and scalable process, fully compatible with the growing demand for greener nutraceutical and pharmaceutical manufacturing.

## 4. Materials and Methods

### 4.1. Materials and Reagents

**Figure 17:**
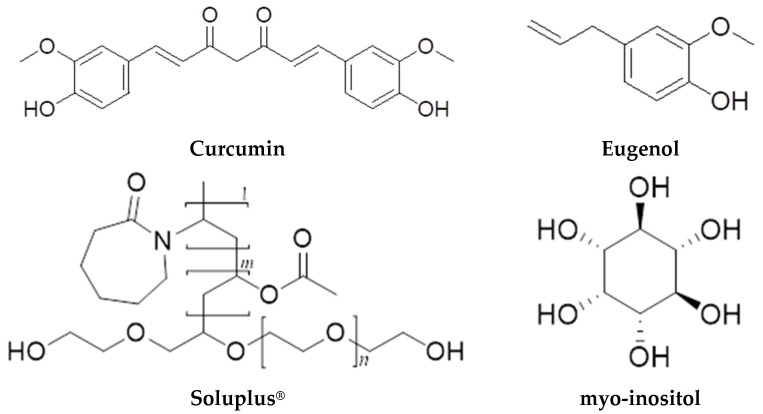
Chemical structures of eugenol, curcumin, Soluplus^®^, and myo-inositol.

Curcumin (Fluorochem, Glossop, UK, 95% purity), Eugenol (AlfaAesar GmbH & Co. KG, Karlsruhe, Germany, 99% purity), Soluplus^®^ (BASF SE, Ludwigshafen, Germany), myo-inositol (AlfaAesar GmbH & Co. KG, Karlsruhe, Germany, 98.0% purity), and Aerosil^®^ 200 (Evonik Operations GmbH, Essen, Germany), were used for preparing the formulations (Figure 17). Distilled water and Ethanol Absolute 99.8%, Certified AR for Analysis (Fisher Chemical™, Pittsburgh, PA, USA), were used as a solvent for the preparation of the feed spray drying dispersions, as ethanol has also been used for the curcumin and eugenol assay.

### 4.2. Preparation of Feed Solutions for Spray Drying

#### 4.2.1. Preparation of Curcumin-Loaded Dispersions for Spray Drying (Summarized in Supplementary Materials, Table S1)

The notations of the formulations contain the percentage of curcumin (C) or/and eugenol (E) masses with respect to the mass of Soluplus^®^, which were used for the preparation of the feed solutions for spray drying. Myo-inositol and Aerosil^®^ 200 are denoted as I and A, respectively.

During ultrasonication (Ultrasonic Cleaner, Isolab, Tense, Shanghai, China), the ethanol solutions were placed in tightly closed glass vials to prevent the evaporation of the solvent.

*Dispersions containing only water or ethanol:*

In total, 4.000 g of Soluplus^®^ and 0.040 g, 0.200 g, and 0.400 g of curcumin for 1%C-S, 5%C-S, and 10%C-S, respectively, were dissolved in 60 mL of water using ultrasound for 20 min. The obtained dispersion was stirred at 400 rpm for 2 h at room temperature, followed by spray drying.

In total, 4.000 g of Soluplus^®^ and 0.400 g curcumin were dissolved in 60 mL of ethanol using ultrasound for 20 min. The obtained transparent solution was spray-dried.

*Dispersions containing water and ethanol*

- *2:1 water/ethanol ratio*

A total of 4.000 g of Soluplus^®^ and 0.200 g and 0.400 g curcumin for 5%C-S and 10%C-S, respectively, were dissolved in 20.0 mL of ethanol (40.0 mL for the total volume of 120 mL), homogenized using ultrasound for 20 min, and slowly dropped into 40.0 mL of water (80.0 mL for the total volume of 120 mL), while being stirred at 400 rpm. The obtained dispersion was stirred at 400 rpm for 2 h at room temperature, followed by spray drying of the micelle solution.

- *5:1 water/ethanol ratio*

A total of 4.000 g of Soluplus^®^ and 0.200 g and 0.400 g curcumin for 5%C-S and 10%C-S, respectively, were dissolved in 20.0 mL of ethanol, homogenized using ultrasound for 20 min, and slowly dropped into 100.0 mL of water, while being stirred at 400 rpm. The obtained dispersion was stirred at 400 rpm for 2 h at room temperature, followed by spray drying.

#### 4.2.2. Preparation of Eugenol-Loaded Dispersions for Spray Drying

A total of 4.000 g of Soluplus^®^ and 0.800 g of eugenol, respectively, were dissolved in 40 mL (SP 10% *w*/*v*) or 60 mL (SP 6.7% *w*/*v*) of water, and the obtained dispersion was stirred at 400 rpm for 8 h at room temperature. Since the dispersion had precipitation in line with our previous research [28], it was left in darkness for 24 h and restirred for 1 h to obtain a stable dispersion without precipitation and spray-dried, followed by spray drying.

A total of 4.000 g of Soluplus^®^ and 0.800 g of eugenol were dissolved in 20.0 mL of ethanol and slowly dropped into 40.0 mL of water, while being stirred at 400 rpm. The obtained dispersion was stirred at 400 rpm for 2 h at room temperature, followed by spray drying.

#### 4.2.3. Preparation of Double-Loaded Curcumin/Eugenol Dispersions for Spray Drying

The double-loaded dispersions were obtained following the same procedure as for 2:1 and 5:1 water/ethanol ratio curcumin-containing dispersions, using the amount of curcumin and eugenol notated in the name of the formulation.

The myo-inositol and Aerosil^®^ 200 containing dispersions were obtained as 4 g of myo-inositol or/and 0.1 g of Aerosil^®^ 200 were added to the double-loaded dispersion.

### 4.3. Spray Drying Conditions

The spray-dried powders were prepared using a Büchi Mini spray dryer B-290 (Büchi Laboretechnik AG, Flawil, Switzerland). Process parameters during spray-drying such as feed rates of 6 mL/min (20% pump), an atomization gas flow rate of 414 L/h (35 mm), an aspiration rate of 35 m^3^/h (aspiration 100%), and an inlet temperature of 80 °C are constant for all formulations. The outlet temperature varies depending on the solvents and was 41 ± 2 °C for the only water containing dispersions and 5:1 for the H_2_O/EtOH ratio, 48 ± 2 °C for the 2:1 H_2_O/EtOH ratio, and 51 ± 2 °C for the 10%C-S powder obtained from ethanol solution. A nozzle with a diameter of 0.7 mm was used. The yield, calculated as the percentage of the collected mass of the spray-dried powders divided by the overall mass of the added compounds in the spray drying feed solutions, was ~20–50% for the formulations only with Soluplus^®^ and ~60% for the formulations containing myo-inositol. The spray-dried powders were stored in the dark in plastic containers at room temperature.

### 4.4. Particle Size in the Water Solution

The hydrodynamic diameter, dispersity, and zeta potential of the spray-dried formulations dissolved in deionized water (1 mg/mL) were measured by using a Zetasizer NanoBrook 90Plus PALS instrument (Holtsville, NY, USA), equipped with a 35 mW red diode laser (λ = 640 nm).

### 4.5. Scanning Electron Microscopy

The SEM images of the powders were obtained using a Hitachi TM4000 Scanning Electron Microscope (Hitachi, Tokyo, Japan) with a BSE detector under a low vacuum. The samples were gold-coated before the registration of the SEM micrographs.

### 4.6. Assay of the Encapsulated Curcumin and Eugenol (Encapsulation Efficiency and Loading Capacity)

Approximately 50.0 mg of the spray-dried samples were dissolved in 10.0 mL of ethanol. The stock solution was filtered with a 0.45 μ syringe filter and then 30 μL of the filtrate was diluted to 2030 μL with ethanol. The absorbance of Soluplus^®^ at a concentration close to the corresponding concentration for the solutions of the samples containing only Soluplus^®^ as excipient at 282 nm and 425 nm was found to be 0.003 and 0.001, respectively. The absorbance was measured at the maximum: 281 nm of Eug and 425 nm of Cur at room temperature using the UV–VIS spectrophotometer, Shimadzu UV-1800 (Shimadzu Corporation, Kyoto, Japan). Since Cur absorbs in the UV region, the amount of Eug in the double-loaded powders was evaluated by subtracting the absorbance of Cur at 281 nm, which was calculated using its concentration found from its absorbance at 425 nm (the absorbance of Eug at 425 nm is 0.00) and its absorptivity at 281 nm. For each sample, three assays were performed, and the average value and standard deviation were calculated (Table S2). The calibration curves of the pure curcumin and eugenol are shown in the Supplementary Materials, Figure S6.

The encapsulation efficiency (%) was calculated in the following way:

EE (%) = (mass of encapsulated curcumin or eugenol)/(Theoretical mass of curcumin/eugenol) × 100,

where encapsulated curcumin/eugenol is the amount of curcumin/eugenol content determined by the absorption of the spray-dried sample in the UV spectrum and theoretical is the added amount of curcumin/eugenol in the solutions for spray drying.

LC (%) = (mass of encapsulated curcumin or eugenol/mass of the polymer) × 100

### 4.7. Fourier-Transform Infrared Spectroscopy

The FTIR spectra of Soluplus^®^, Curcumin, Eugenol, and selected spray-dried Cur/Eug-loaded powders were recorded with an FTIR spectrometer (IRAffinity-1, Shimadzu, Kyoto, Japan), equipped with ATR, in the range 4000–600 cm^−1^. Signal averages were obtained at a resolution of 4 cm^−1^.

### 4.8. Differential Scanning Calorimetry

DSC thermograms were obtained using Q200 TA Instruments, New Castle, DE, USA, in the interval of 25–285 °C at a rate of 5 °C/min under a nitrogen atmosphere at a flow rate of 50 mL/min. The samples were sealed in Tzero Aluminium pans, and for the pure eugenol, a Tzero Hermetic pan (TA Instruments, New Castle, DE, USA) was used.

### 4.9. X-Ray Diffraction

Wide-angle X-ray diffraction (WAXD) patterns were obtained on a Bruker D8 Advance ECO diffractometer (Bruker, Billerica, MA, USA), operating at 40 kV and 25 mA in the Bragg–Brentano geometry with Ni-filtered Cu Kα radiation and a LynxEye-XE detector (Bruker, Billerica, MA, USA) over the 2θ range of 5–80°, with a scanning rate of 0.02° s^−1^.

### 4.10. Dissolution of Curcumin and Eugenol from the Spray-Dried Powders

The release profiles were estimated using the PJ-3 Tablet Four-Usage Tester, SaintyCo, Rui’an, China (Apparatus 2-Rotating Paddle). Then, 0.500 g of the powders were placed in 500.0 mL distilled H_2_O and stirred at 25 °C with a speed of 100 rpm. Samples of 1.0 mL were withdrawn at 5, 10, 15, 20, 30, 45, 60, and 90 min and diluted with ethanol to 10.0 mL in a volumetric flask. The amount of curcumin and eugenol in the dissolved powder was evaluated using UV spectroscopy and expressed as a percentage of the curcumin/eugenol content in the formulations.

## 5. Conclusions

This study presents a novel and scalable green technology for co-encapsulating hydrophobic bioactive compounds, specifically curcumin and eugenol, significantly enhancing their aqueous solubility and dispersibility. The optimized spray drying process successfully addressed key formulation challenges, including the thermoplastic nature of Soluplus^®^ and the volatility of eugenol. The amorphous dispersion of curcumin and eugenol within the Soluplus^®^ matrix, indicated by the absence of crystalline diffraction peaks, likely involves hydrogen bonding and hydrophobic interactions, significantly enhancing solubility and stability. Additionally, the incorporation of myo-inositol markedly improved powder dispersibility, reduced particle aggregation, and served as an effective crystallization inhibitor. The developed formulations are particularly suited for practical applications such as dietary supplements, functional beverages, and pharmaceutical excipients, enabling effective oral delivery of hydrophobic therapeutic agents. Further studies, including detailed pharmacokinetic evaluations, bioavailability comparisons with existing delivery systems, and stability testing under various storage conditions, will be crucial to fully establish clinical and commercial applicability.

## Figures and Tables

**Table 1:** Summary of the curcumin (Cur) and Soluplus^®^ (SP) content in the solutions for spray drying, volume and ratio of the solvents, hydrodynamic diameter (D_h_), zeta potential, and dispersity index (DI) of the obtained Cur-loaded SP-nanoparticles.

Formulation	Cur, g	SP, g	H_2_O:EtOH Ratio	H_2_O + EtOH, mL	Yield, %	Conc Polymer Solution, %	D_h_ ^1^, nm	Zeta Potential ^1^, mV	DI ^1,2^
S	0.000	4	1:0	60.0	27.1	6.7	54 ± 2	−3.00 ± 0.8	0.06 ± 0.011
1%C-S	0.040	4	1:0	60.0	26.4	6.7	53 ± 2	−3.80 ± 1.1	0.09 ± 0.023
5%C-S	0.200	4	1:0	60.0	22.5	6.7	Precipitation		
5%C-S	0.200	4	2:1	60.0	24.7	6.7	57 ± 1	−4.17 ± 1.3	0.07 ± 0.034
10%C-S	0.400	4	1:0	60.0	25.2	6.7	Precipitation		
10%C-S	0.400	4	0:1	60.0	52.5	6.7	74, 204 ± 2	−5.25 ± 1.8	0.24 ± 0.025
10%C-S	0.400	4	2:1	60.0	31.8	6.7	90 ± 1	−6.50 ± 1.5	0.10 ± 0.022
10%C-S	0.400	4	2:1	120.0	56.6	3.3	100 ± 3	−2.26 ± 1.4	0.18 ± 0.006
10%C-S	0.400	4	5:1	120.0	52.5	3.3	62 ± 1	−5.70 ± 1.9	0.08 ± 0.043

^1^ Values are expressed as mean ± standard deviation. ^2^ *p* < 0.05 between different systems.

**Table 2:** Summary of the eugenol (Eug) and Soluplus^®^ (SP) content in the solutions for spray drying, volume and ratio of the solvents, hydrodynamic diameter (D_h_), zeta potential, and dispersity index (DI) of the obtained Eug-loaded SP-nanoparticles.

Formulation	Eug, g	SP, g	H_2_O:EtOH Ratio	H_2_O + EtOH mL	Yield, %	Conc Polymer Solution%	Dh ^2^, nm	Zeta Potential ^2^, mV	DI ^2,3^
5%E-S ^1^	0.200	4	1:0	40.0	47.1	10.0	51 ± 3	−4.6 ± 0.7	0.04 ± 0.011
10%E-S ^1^	0.400	4	1:0	40.0	45.9	10.0	52 ± 3	−4.5 ± 1.2	0.09 ± 0.008
15%E-S ^1^	0.600	4	1:0	40.0	44.1	10.0	54 ± 2	−4.3 ± 1.6	0.04 ± 0.024
20%E-S	0.800	4	1:0	40.0	43.4	10.0	59 ± 3	−5.7 ± 0.9	0.10 ± 0.011
20%E-S	0.800	4	1:0	60.0	37.0	6.7	56 ± 2	−4.9 ± 1.1	0.09 ± 0.018
20%E-S	0.800	4	2:1	60.0	12.2	6.7	108 ± 6	−2.9 ± 1.8	0.10 ± 0.012

^1^ The powders were obtained in our previous study [28]. ^2^ Values are expressed as mean ± standard deviation. ^3^ *p* < 0.05 between different systems.

**Table 3:** Summary of the curcumin (Cur), eugenol (Eug), and Soluplus^®^ (SP) content in the solutions for spray drying, volume and ratio of the solvents, hydrodynamic diameter (Dh), zeta potential, and dispersity index (DI) of the obtained double-loaded SP-nanoparticles.

Formulation	Cur, g	Eug, g	SP, g	H_2_O:EtOH Ratio	H_2_O + EtOH mL	Yield, %	Conc Polymer Solution%	Dh ^1^, nm	Zeta Potential ^1^, mV	DI ^1,2^
5%C,5%E-S	0.200	0.200	4	2:1	120.0	41.4	3.3	56 ± 3	−2.4 ± 1.7	0.08 ± 0.008
5%C,10%E-S	0.200	0.400	4	2:1	120.0	37.0	3.3	73 ± 2	−3.2 ± 1.4	0.12 ± 0.008
5%C,20%E-S	0.200	0.800	4	2:1	120.0	23.4	3.3	78 ± 1	−7.2 ± 0.7	0.13 ± 0.015
10%C,5%E-S	0.400	0.200	4	2:1	120.0	49.0	3.3	60 ± 3	−4.1 ± 1.1	0.07 ± 0.017
10%C,10%E-S	0.400	0.400	4	2:1	120.0	46.2	3.3	66 ± 2	−5.4 ± 1.3	0.14 ± 0.011
10%C,15%E-S	0.400	0.600	4	2:1	120.0	36.7	3.3	69, 265 ± 4	−7.7 ± 1.5	0.24 ± 0.017
10%C,20%E-S	0.400	0.800	4	2:1	120.0	28.7	3.3	152,828 ± 3	−9.6 ± 1.8	0.27 ± 0.016
10%C,20%E-S	0.400	0.800	4	5:1	120.0	37.9	3.3	127 ± 2	−6.9 ± 1.8	0.16 ± 0.029
5%C,5%E-S-I	0.200	0.200	4	5:1	120.0	57.0	3.3	61 ± 3	−6.7 ± 1.6	0.14 ± 0.018
10%C,15%E-S-I	0.400	0.600	4	5:1	120.0	58.5	3.3	89 ± 3	−12.5 ± 2.1	0.20 ± 0.010
10%C,20%E-S-I-A	0.400	0.800	4	5:1	120.0	56.3	3.3	132, 373, 1235 ± 6	−17.7 ± 2.3	0.273 ± 0.009
10%C,20%E-S-I	0.400	0.800	4	2:1	120.0	59.1	3.3	84, 292 ± 5	−8.8 ± 2.4	0.275 ± 0.013
10%C,20%E-S-I-A	0.400	0.800	4	2:1	120.0	55.7	3.3	230 ± 2	−15.4 ± 1.9	0.225 ± 0.040

^1^ Values are expressed as mean ± standard deviation. ^2^ *p* < 0.05 between different systems.

## Data Availability

Data is contained in the paper.

## Supplementary Materials

The following supporting information can be downloaded at https://www.mdpi.com/article/10.3390/ph18070944/s1: Figure S1: DLS measurements of SP in water and water/ethanol mixtures; Figure S2: Hydrodynamic diameter of spray-dried SP polymeric micelles; Figure S3: SEM micrographs of selected powders; Figure S4: DSC thermograms of the pure SP and 20%E-S and 10/C,20%E-S powders; Figure S5: HPLC chromatograms of pure curcumin and eugenol, and selected powders; Figure S6: Calibration curves of curcumin and eugenol; Table S1: Composition summary of spray-drying feed dispersions; Table S2: Encapsulation efficiency, EE%, and standard deviation of the single- and double-loaded formulations.
