# Supplementary material for: Water-Soluble Formulations of Curcumin and Eugenol Produced by Spray Drying
Source: Pharmaceuticals (Basel). 2025 Jun 23;18(7):944. doi: 10.3390/ph18070944 (PMC12300477; doi:10.3390/ph18070944)
Supplement: Supplementary file 1 [file pharmaceuticals-18-00944-s001.zip › pharmaceuticals-3687602-supplementary.pdf]

Supplementary Materials

Preparation and characterization of water-soluble formulations of Curcumin and Eugenol by Spray Drying method

Iskra Z. Koleva,[a] Katya Kamenova,[b] Petar D. Petrov,[b] Christo T. Tzachev[a]

[a]*Faculty of Chemistry and Pharmacy, Sofia University "St. Kliment Ohridski",*

1 J. Bourchier Blvd., 1164 Sofia, Bulgaria

[b]*Institute of Polymers, Bulgarian Academy of Sciences, "Akad. G. Bonchev" Street., bl. 103A, 1113 Sofia, Bulgaria*

List of content:

Figure S1. DLS measurements of SP in water and water/ethanol mixtures.

Figure S2. Hydrodynamic diameter of spray-dried SP polymeric micelles.

Figure S3. SEM micrographs of selected powders.

Figure S4. DSC thermograms of the pure SP and 20%E-S and 10C,20%E-S powders.

Figure S5. HPLC chromatograms of pure curcumin and eugenol, and selected powders.

Figure S6. Calibration curves of curcumin and eugenol.

Table S1. Composition summary of spray-drying feed dispersions

Table S2. Encapsulation efficiency, EE% and standard deviation of the single- and double-loaded formulations.

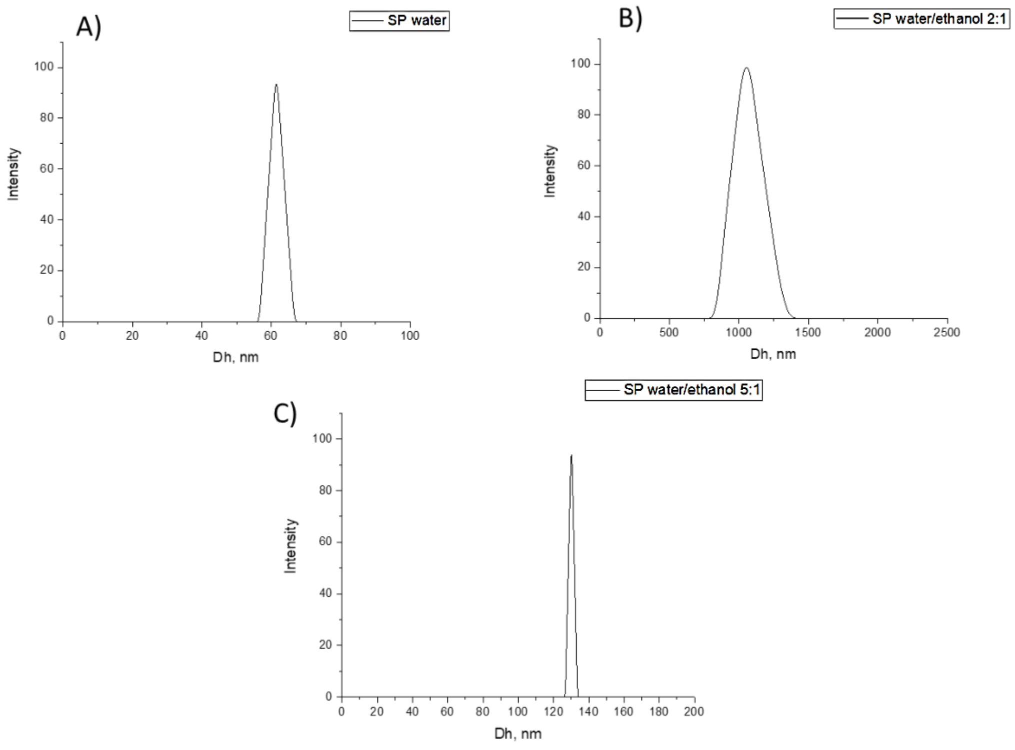

Figure S1. DLS measurements of SP in water (A) and water/ethanol mixtures at 2:1 (B) and 5:1 (C) ratios.

SP powder was prepared by dissolving 4 g of Soluplus in water (120 mL), followed by spray drying of the micelle solution. The yield of SP solid particles was 30%. After dissolving in water, the nanocarriers exhibited a small size of 54 nm and a negative zeta potential of -3 mV (Figure S2).

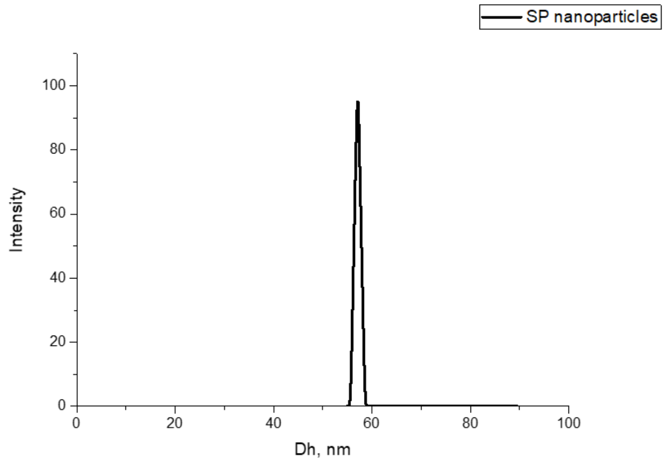

Figure S2. Hydrodynamic diameter of spray-dried SP polymeric micelles.

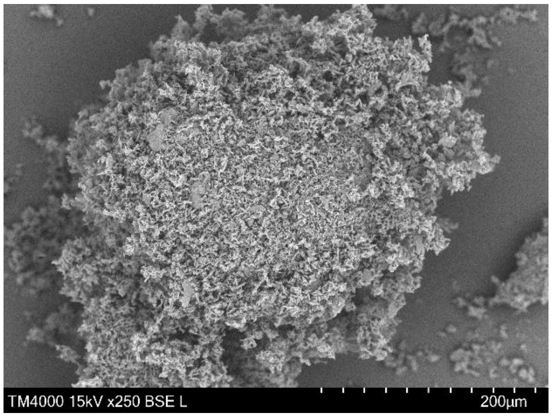

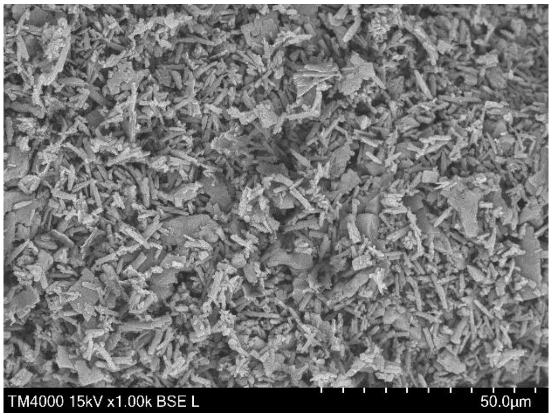

Pure curcumin

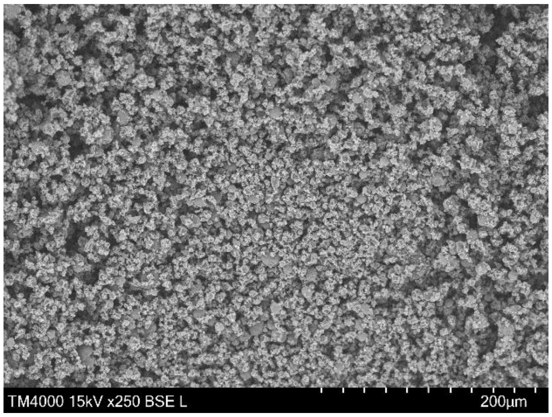

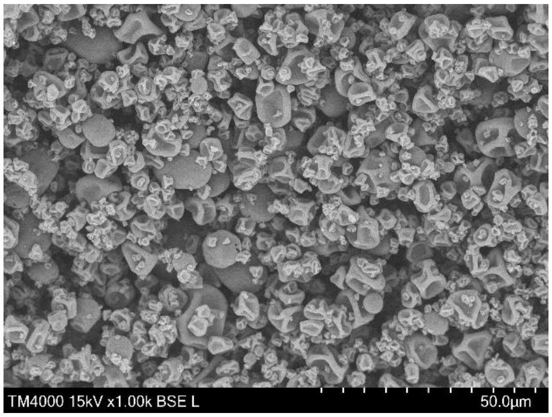

1%C-S from only H_2O dispersion

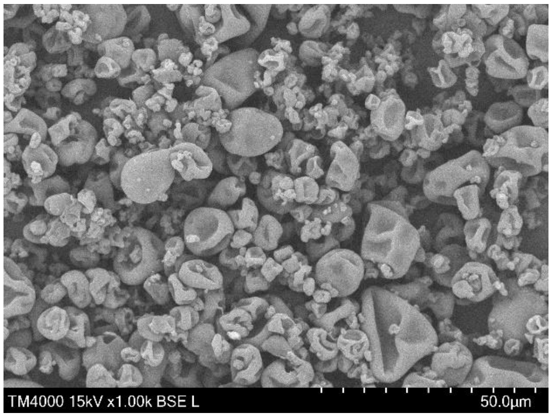

5%C-S H_2O:EtOH 2:1

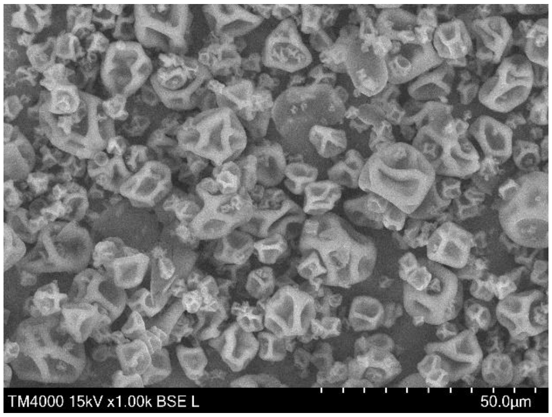

10%C-S from only H_2O dispersion

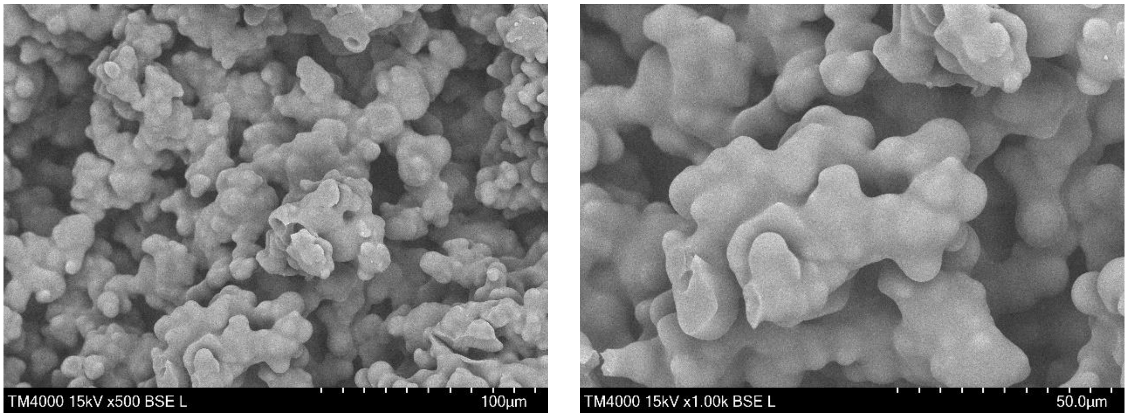

20%E-S H_2O:EtOH 2:1

Figure S3. SEM micrographs of selected powders.

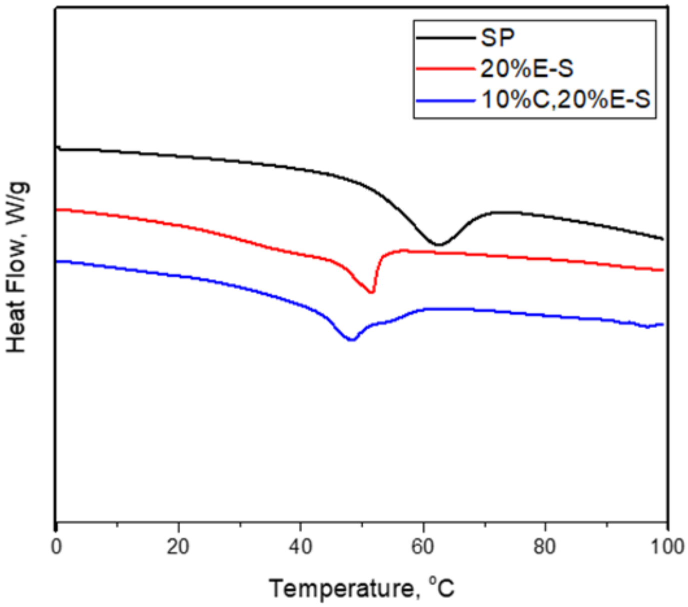

Figure S4. DSC thermograms of the pure SP and 20%E-S and 10C,20%E-S powders.

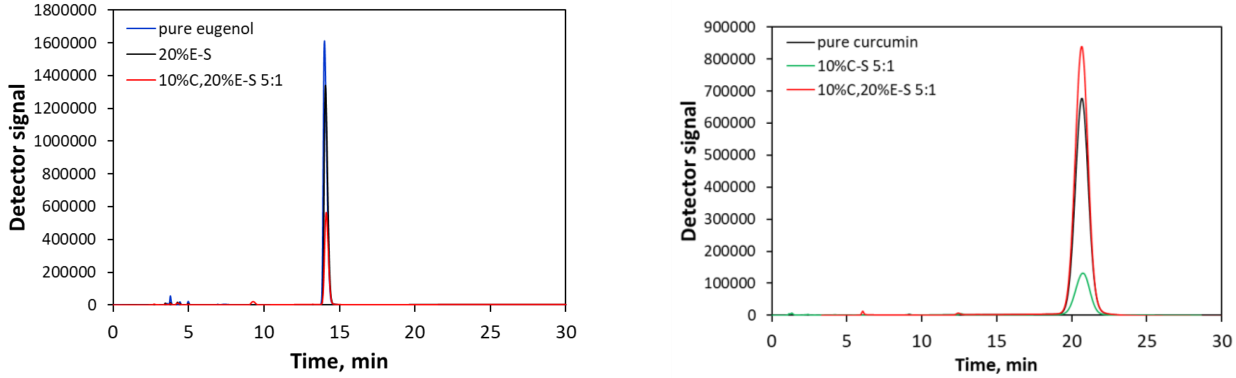

Figure S5. HPLC chromatograms of pure curcumin and eugenol, and selected powders.

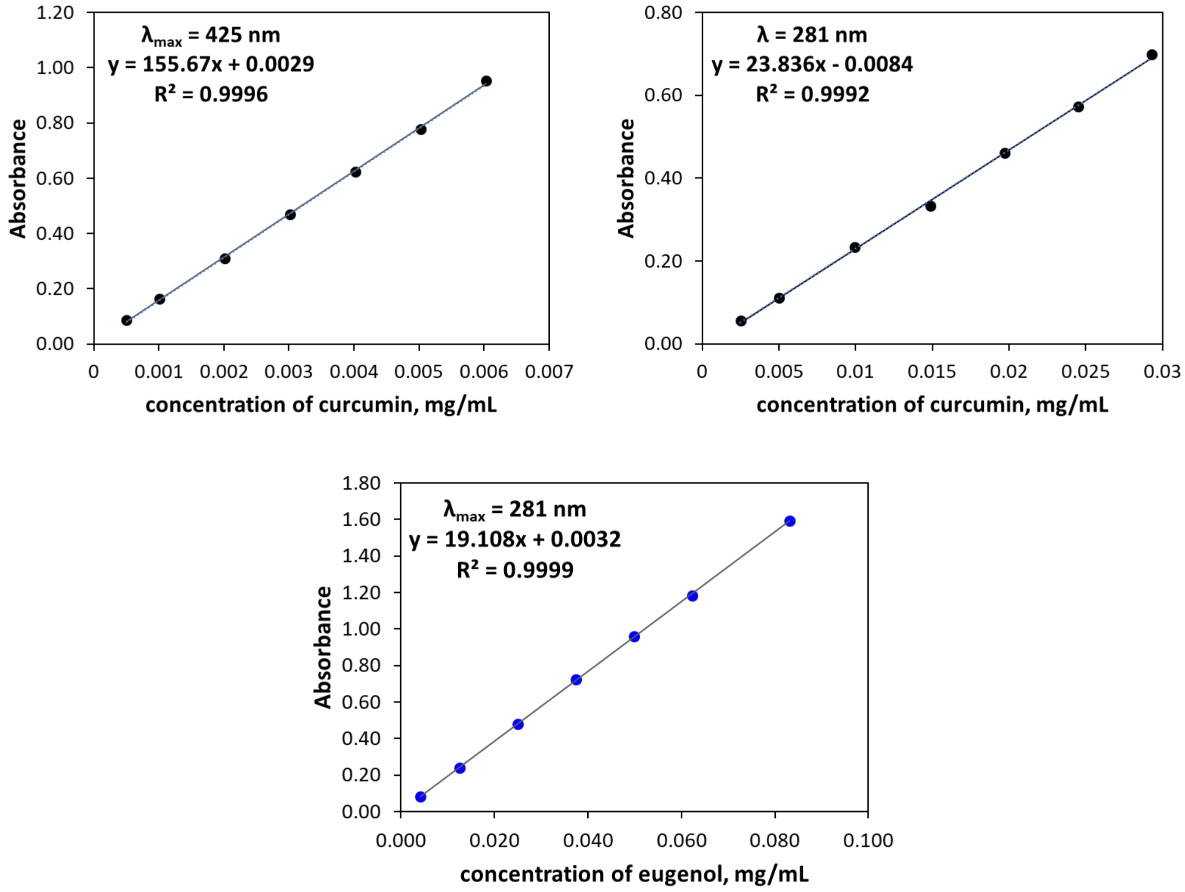

Figure S6. Calibration curves of curcumin and eugenol.

Table S1. Composition summary of spray-drying feed dispersions.

Formulation	Cur, g	Eug, g	SP, g	H_2O, mL	EtOH, mL	H_2O:EtOH ratio	H_2O+EtOH, mL	Conc. polymer solution, %
S	0.000	0.000	4	60	0	1:0	60.0	6.7
1%C-S	0.040	–	4	60	0	1:0	60.0	6.7
5%C-S	0.200	–	4	**60**	**0**	1:0	60.0	6.7
5%C-S	0.200	–	4	40	20	2:1	60.0	6.7
10%C-S	0.400	–	4	60	0	1:0	60.0	6.7
10%C-S	0.400	–	4	0	60	0:1	60.0	6.7
10%C-S	0.400	–	4	40	20	2:1	60.0	6.7
10%C-S	0.400	–	4	80	40	2:1	120.0	3.3
10%C-S	0.400	–	4	100	20	5:1	120.0	3.3
5%E-S[a]	–	0.200	4	40	0	1:0	40.0	10.0
10%E-S[a]	–	0.400	4	40	0	1:0	40.0	10.0
15%E-S[a]	–	0.600	4	40	0	1:0	40.0	10.0
20%E-S	–	0.800	4	40	0	1:0	40.0	10.0
20%E-S	–	0.800	4	60	0	1:0	60.0	6.7
20%E-S	–	0.800	4	40	20	2:1	60.0	6.7
5%C,5%E-S	0.200	0.200	4	80	40	2:1	120.0	3.3
5%C,10%E-S	0.200	0.400	4	80	40	2:1	120.0	3.3
5%C,20%E-S	0.200	0.800	4	80	40	2:1	120.0	3.3
10%C,5%E-S	0.400	0.200	4	80	40	2:1	120.0	3.3
10%C,10%E-S	0.400	0.400	4	80	40	2:1	120.0	3.3
10%C,15%E-S	0.400	0.600	4	80	40	2:1	120.0	3.3
10%C,20%E-S	0.400	0.800	4	80	40	2:1	120.0	3.3
10%C,20%E-S	0.400	0.800	4	100	20	5:1	120.0	3.3
5%C,5%E-S-I	0.200	0.200	4	100	20	5:1	120.0	3.3
10%C,15%E-S-I	0.400	0.600	4	100	20	5:1	120.0	3.3
10%C,20%E-S-I-A	0.400	0.800	4	100	20	5:1	120.0	3.3
10%C,20%E-S-I	0.400	0.800	4	80	40	2:1	120.0	3.3
10%C,20%E-S-I-A	0.400	0.800	4	80	40	2:1	120.0	3.3

[a] The powders were obtained in our previous study [Koleva, I.Z.; Tzachev, C.T. Efficient Improvement of Eugenol Water Solubility by Spray Drying Encapsulation in Soluplus® and Lutrol F 127. *Pharmaceuticals* **2024**, 17, 1156. https://doi.org/10.3390/ph17091156]

Table S2. Encapsulation efficiency, EE% and standard deviation of the single- and double-loaded formulations.

Formulation	Cur, EE%±SD	Eug, EE%±SD
1%C-S (H_2O; 6.7 mass%)	96.1 ± 2.0	–
5%C-S (H_2O/EtOH 2:1; 6.7 mass%)	97.8 ± 4.0	–
10%C-S (EtOH 6.7 mass%)	91.9 ± 4.8	–
10%C-S (H_2O/EtOH 2:1; 6.7 mass%)	93.5 ± 1.7	–
10%C-S (H_2O/EtOH 2:1; 3.3 mass%)	97.6 ± 3.5	–
10%C-S (H_2O/EtOH 5:1; 3.3 mass%)	98.4 ± 2.5	–
5%E-S (10 mass%)	–	98.2 ± 1.9
10%E-S (10 mass%)	–	92.7 ± 1.4
15%E-S (10 mass%)	–	90.9 ± 2.1
20%E-S (10 mass%	–	76.3 ± 1.5
20%E-S (6.7 mass%)	–	76.0 ± 2.9
20%E-S (H_2O/EtOH 2:1; 6.7 mass%)	–	10.9 ± 1.5
5%C,5%E-S (H_2O/EtOH 2:1)	97.2 ± 2.6	93.6 ± 2.0
5%C,10%E-S (H_2O/EtOH 2:1)	96.9 ± 3.0	86.2 ± 4.2
5%C,20%E-S (H_2O/EtOH 2:1)	96.2 ± 0.5	83.4 ± 1.7
10%C,5%E-S (H_2O/EtOH 2:1)	97.1 ± 3.9	91.5 ± 3.5
10%C,10%E-S (H_2O/EtOH 2:1)	95.9 ± 1.3	83.3 ± 0.8
10%C,15%E-S (H_2O/EtOH 2:1)	95.8 ± 4.0	82.7 ± 3.4
10%C,20%E-S (H_2O/EtOH 2:1)	96.1 ± 2.2	79.9 ± 3.0
10%C,20%E-S (H_2O/EtOH 5:1)	96.5 ± 2.3	79.8 ± 2.7
5%C,5%E-S-I (H_2O/EtOH 2:1)	96.0 ± 3.0	91.5 ± 3.3
10%C,15%E-S-I (H_2O/EtOH 2:1)	89.7 ± 4.0	81.2 ± 2.9
10%C,20%E-S-I-A (H_2O/EtOH 2:1)	93.3 ± 3.9	72.4 ± 4.6
10%C,20%E-S-I (H_2O/EtOH 5:1)	88.9 ± 3.4	96.5 ± 3.6
10%C,20%E-S-I-A (H_2O/EtOH 5:1)	93.3 ± 1.2	74.4 ± 3.9
